# Testing the ecological consequences of evolutionary change using elements

**DOI:** 10.1002/ece3.950

**Published:** 2014-01-15

**Authors:** Punidan D Jeyasingh, Rickey D Cothran, Michael Tobler

**Affiliations:** 1 Department of Zoology, Oklahoma State UniversityStillwater, Oklahoma, 74078; 2 Department of Biological Sciences and Pymatuning Laboratory of Ecology, University of PittsburghPittsburgh, Pennsylvania, 15260

**Keywords:** Consumer-driven nutrient recycling, eco-evo-eco loop, eco-evolutionary dynamics, ecological stoichiometry, intraspecific variation, ionomics, nutrient excretion, phenotypic evolution

## Abstract

Understanding the ecological consequences of evolutionary change is a central challenge in contemporary biology. We propose a framework based on the ˜25 elements represented in biology, which can serve as a conduit for a general exploration of poorly understood evolution-to-ecology links. In this framework, known as ecological stoichiometry, the quantity of elements in the inorganic realm is a fundamental environment, while the flow of elements from the abiotic to the biotic realm is due to the action of genomes, with the unused elements excreted back into the inorganic realm affecting ecological processes at higher levels of organization. Ecological stoichiometry purposefully assumes distinct elemental composition of species, enabling powerful predictions about the ecological functions of species. However, this assumption results in a simplified view of the evolutionary mechanisms underlying diversification in the elemental composition of species. Recent research indicates substantial intraspecific variation in elemental composition and associated ecological functions such as nutrient excretion. We posit that attention to intraspecific variation in elemental composition will facilitate a synthesis of stoichiometric information in light of population genetics theory for a rigorous exploration of the ecological consequences of evolutionary change.

## Ecological Relevance of Evolutionary Change

Work linking ecology and evolution has primarily focused on how ecology affects evolutionary change. Ecology determines both the direction and magnitude of selection on phenotypes (Endler 1986). The opposing direction – how evolution shapes ecology – has received much less attention, because evolution was thought to be too slow to affect ecological processes (Schoener 2011). Rapid evolutionary change, however, is common (e.g., Hendry et al. 2000; Hairston et al. 2005), and an evo-eco link is established when such change alters the environment experienced by individuals. Moreover, if the resulting ecological change subsequently affects fitness, an eco-evo-eco feedback loop can be generated that results in rapid and often cyclical evolutionary change (e.g., Yoshida et al. 2003; Fussmann et al. 2007; Turcotte et al. 2011; Becks et al. 2012). Such feedbacks were first postulated over 50 years ago (Pimentel 1961), although their ecological importance has only recently been appreciated and have rarely been tested in natural systems (Post and Palkovacs 2009; Schoener 2011; Reznick 2013).

Because evolutionary change in a trait often occurs simultaneously with change in environmental parameters that interact to affect ecology, Hairston et al. (2005) presented a framework to disentangle the relative contributions of genetic and environmental change in driving ecologically relevant evolutionary shifts. They drew upon rigorous, long-term datasets, for example, on Darwin's finches (Grant and Grant 2002), to demonstrate that evolved shifts in beak size and a key environmental parameter (mean seed size) interact to determine growth rate of the finch population, a key demographic parameter that should impact local ecological processes. Ellner et al. (2011) subsequently included nonheritable phenotypic changes using the Price equation (Price 1972), to better estimate the ecological importance of evolution. Specifically, they used data from Hairston et al. (2001), with juvenile growth rate of *Daphnia* as an evolving trait and food quality as the source of selection, to show that a significant percentage of change in adult body mass (24%) was driven by evolution. Rapid evolutionary change in adult body size of daphniids is predicted to have strong ecological consequences because body size affects population growth rate through correlated changes in fecundity and predation risk (e.g., Hall et al. 1976).

Such conceptual progress (see also Collins and Gardner 2009) has been useful to understand the ecological consequences of evolutionary change, particularly in traits closely linked to demographics (e.g., growth rate) that undoubtedly are ecologically relevant. However, focusing on the evolution of traits (e.g., growth rate, beak size) to understand links between evolution and ecology is wrought with challenges, because apparently simple selective regimes that can explain a wide variety of phenotypic variation often prove to be notoriously complex (Grether et al. 2001). Evolutionary biologists are beginning to embrace environmental complexity to understand phenotypic evolution in response to multifarious selection (e.g., Kaeuffer et al. 2012). While traits exhibiting rapid evolution have been a prerequisite in studying evolution-to-ecology links, attention to such traits introduces biases that can result in failure to identify an ecological response when accommodative or correlated evolution in other traits (e.g., Brodie 1992) affects a different aspect of ecology. High-throughput phenotyping will allow for more robust inferences about trait evolution and its importance in ecology; however, such technologies are not widely applied in evolutionary ecology (e.g., Houle et al. 2010; NSF-USDA 2011). Phenomics has been applied, notably in agronomy, to understand traits such as drought resistance and nutrient efficiency using high-throughput imaging and analyses (e.g., Yang et al. 2013). Such photonics-based technologies are useful to create ever-finer maps linking the genotype and phenotype, but whether they can be used effectively to characterize the phenome of mobile organisms remains a question. Even if we can overcome these challenges, quantifying the ecological consequences of evolutionary change in multiple traits expressed by an individual would still be a daunting task.

## Defining the Elemental Phenotype

**Figure 1:**
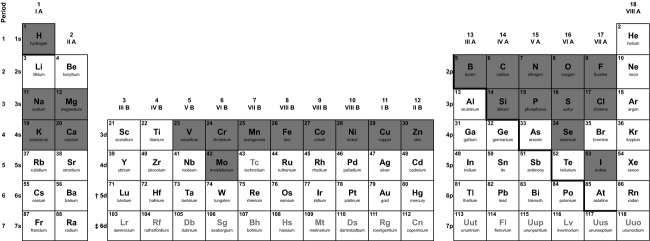
Periodic table indicating the elements represented in biology (grey squares). After Frausto da Silva and Williams (1991).

We propose that taking an elemental view of phenotypic variation can simplify the daunting task of understanding the ecological importance of phenotypic evolution. While high-throughput phenomics is in its infancy, Salt et al. (2008) proposed high-throughput ionomics, using mass spectrometry, as a sophisticated alternative that is applicable to all taxa. Advances in mass spectroscopy (e.g., inductively coupled plasma, ICP-MS) enable rapid quantification of the majority of the ˜25 elements represented in biology (Fig 1). Each of these elements play key roles in the fundamental biochemistry of life ranging from storage of genetic information to orchestration of metabolic processes, and the expression of anatomical and morphological traits (see Frausto da Silva and Williams 1991). Such technological advancements enable the rapid quantification of phenotypes in the same currency used to study key ecological processes such as nutrient recycling and enable a mechanistic exploration of the links between evolutionary change in populations and associated shifts in ecological function based on first principles (i.e., mass balance). We propose to use the elemental composition of an organism, the flux of the ˜25 elements between an organism and its environment (i.e. acquisition, excretion), and distributional dynamics of elements within the organism (i.e. assimilation, allocation) as an abstraction of classic phenotypes that is applicable to all taxa. Whereas the *classic phenotype* is a unique combination of traits exhibited by an individual that includes biochemical, developmental, physiological, behavioral, morphological, and life-history traits, the *elemental phenotype* is defined as the composition, acquisition, assimilation, allocation, and excretion (CAAA&E) of the ˜25 elements of an individual. It follows that CAAA&E of each element is analogous to a classic trait. Both the classic and the elemental views of a phenotype are complementary. The elemental phenotype of an individual represents a composite phenotype that is fundamentally linked to the expression of the classic phenotype. Recent methodological advancements enable high-throughput quantification of elemental composition and dynamics at multiple levels of organization and can capture the elemental signatures of variation and evolution in classic phenotypes (Box 1). As such, these high-throughput methods enable reliable quantification of the ˜25 fundamental traits that represent an individual. While we realize that the resulting 25 × 25 G-matrix is still complex, these 25 elemental traits can be reliably (and rapidly) quantified compared to traits at higher levels of organization (e.g., molecular, organ) and are applicable to all taxa. Furthermore, because the origin of these 25 elements in an individual is the environment, we can use the same high-throughput methods to reliably quantify environmental parameters and consequently the environment's contribution to phenotypic evolution (West-Eberhard 2003), at the elemental level of organization.

**Box 1. *High throughput methods used to quantify elements at multiple levels of organization.***

High-throughput methods to quantify content, acquisition, assimilation, allocation, and excretion (CAAA&E) of elements at various levels of organization. These methods can be integrated with high-throughput genomic methods enabling a mechanistic exploration of eco-evo-eco links using elements. We recognize it is currently difficult to simultaneously deploy all of the following tools. Nevertheless, it is evident that high-throughput technologies and large databases already exist to rigorously answer the questions posed in Fig. 4.

**Intraindividual**

Often referred to as elemental mapping several methods exist for characterizing the Distribution of elements within a cell tissue or individual. Laser ablation inductively coupled plasma mass spectrometry (LA-ICP-MS) and scanning micro-X-ray fluorescence spectrometry (micro-XRF) are the most commonly used methodologies. Gholap et al. (2010) compared both methods to generate elemental maps of *Daphnia magna* and found that detection power of each method varied depending on the element; thus, they should be used in concert. Similarly, Scott and Ritchie (2009) coupled micro-XRF-based data and simulation modeling to generate an accurate map of elemental distribution in diatom cells. Such technologies are rapidly advancing and produce precise maps of the distribution of elements at a variety of scales (e.g., from the nanometer to centimeter scales), which is an effective tool to study elemental allocation within individuals.

**Organismal**

While elemental mapping is useful to get snapshots of elemental distribution at various scales, it is not conducive to capturing the temporal dynamics of element use (i.e., AAA & E) that is central to the proposed elemental framework linking ecology and evolution. Radio- or stable isotopes of biologically important elements are ideal tools to precisely quantify the dynamics in element use at the cellular as well as individual levels (e.g., Wolfe 1992). Such isotopes have been used to quantify rates of acquisition and assimilation of various elements (e.g., DeMott et al. 1998) and the allocation rates of elements to specific tissues (e.g., Bearhop et al. 2002). Note that by applying mass balance rules, such studies can estimate excretion of elements. Moreover, several studies have used isotopes to directly measure excretion rates of elements (e.g., Fowler et al. 1975). Such tools when used in concert with high-throughput methods such as LA-ICP-MS should be powerful in isolating the sources of variation in the acquisition, assimilation, allocation, and excretion of elements.

**Population**

The fact that microbial, plant, and animal biologists have generated population-level profiles of all the major biologically relevant ions (i.e., ionomics) is testament to genetic variation in how individuals use elements. Indeed, databases with ionomic and genomic information for plant genotypes already exist (e.g., Colmsee et al. 2012). While more widely applied to plants (Salt et al. 2008; Baxter 2010), ionomics has also been used on heterotrophic consumers such as humans to identify the elemental basis of genetically based clinical conditions such as bipolar disorder (Sussulini et al. 2011). Such approaches coupled with robust breeding designs have the power to identify the genetic basis and the effects of environmental conditions (i.e., supply) in determining the elemental phenotype of organisms (e.g., Ardini et al. 2013). Importantly, the same approaches can also be used to understand the genetic basis of excretion of elements (Sasikala-Appukuttan et al. 2010).

**Environment**

Mapping the spatiotemporal dynamics in elemental supply will be key to understanding the ecological relevance of variation in excretion. Several collaborative efforts such as the Nutrient Network (http://www.nutnet.umn.edu/), Ionomics Atlas (http://www.ionomicshub.org/ionomicsatlas), and Geotraces (http://www.geotraces.org/) are producing rapidly growing databases with information on environmental supply of various elements. Methods used to quantify elemental supply in ecosystems range from traditional inorganic chemistry to the X-ray and mass-spectrometry-based methods discussed above. While fine-scale maps of elemental supply do not presently exist, even coarse geochemical information will allow powerful inferences about the ecology and evolution of ecosystems, as elegantly expounded by Orians and Milewski (2007). The geochemical information available in these databases will enhance our ability to link ecology and evolution using the elemental framework. Computational tools such as geographical information systems (GIS)-based niche modeling that link environmental parameters to understand evolutionary patterns are readily available (Kozak et al. 2008).

## Using the Elemental Phenotype to Explore the Ecological Relevance of Evolutionary Dynamics

**Figure 2:**
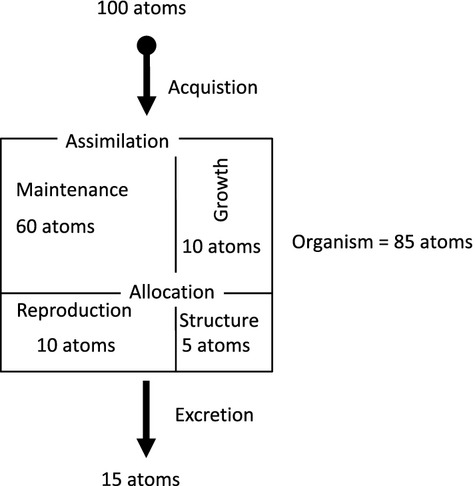
Schematic representation of the mass-balance-based elemental framework proposed for a single element and a single genotype. Based on first principles, the concentration of an element in an individual is a function of acquisition, assimilation, and allocation of the element. The excretion (i.e., unused or waste) of this element can be predicted applying mass balance. Note that this framework applies to all of the ˜25 elements represented in biology.

Alfred Lotka (1925) posited that to understand the evolutionary play on the ecological stage, equal attention should be paid to both, because they are all made of the same materials (i.e., atoms). Indeed, these materials are continuously exchanged between the biotic and abiotic realm, fundamentally coupling ecology and evolution. It follows that the supply of the ˜25 elements involved in biology represents a fundamental, yet quantifiable environment. The elemental composition of an individual is determined by the acquisition of elements from the environment, and assimilation and allocation within the individual. The by-products (e.g., metabolic waste) and unused materials are returned back to the environment representing a fundamental ecological function (Sterner and Elser 2002; Fig. 2). Sterner et al. (1992) championed such an approach by stating “biogeochemical studies often (for whatever reason) pay little attention to interspecific variations in constituent species and in their individual dynamics, concentrating instead on coarser categories such as trophic levels.” Attention to interspecific variation in elemental composition triggered a vibrant and rewarding area of ecological research over the last two decades that integrated diverse biological disciplines (Elser and Hamilton 2007).

**Figure 3:**
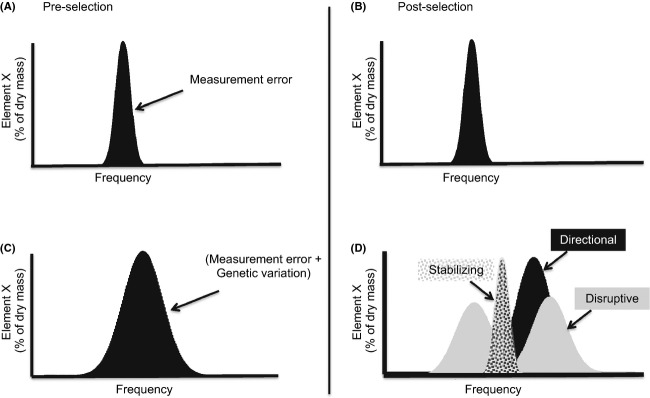
Intraspecific variation in somatic stoichiometry of consumers in the current and proposed stoichiometric frameworks. (A and B) The current framework is not amenable to evolutionary arguments, because under similar supply stoichiometry (i.e., common garden), individuals of similar age (i.e., controlling for ontogenetic effects) are not visible to selection. (C and D) The proposed framework recognizes genetic variation, even after controlling for effects of supply and ontogeny, on which selection can act and potentially alter elemental composition and associated ecological functions.

Nevertheless, ecological stoichiometry has paid little attention to intraspecific variation in elemental composition, even in the three major elements (i.e., carbon, nitrogen, phosphorus) that are often quantified by ecosystem ecologists. This neglect of within-species variation in somatic elemental composition, although useful for understanding higher order ecology, hinders the formal integration of ecological stoichiometry and evolutionary biology (Fig. 3A). Although there have been strategic attempts to incorporate intraspecific variation into ecological stoichiometry (e.g., Villar-Argaiz et al. 2002), to our knowledge, genetic sources of such variation have not been considered. Hence, a key by-product of the current stoichiometric framework is that the elemental phenotype cannot evolve (Fig. 3B). In fact, we know little about the evolutionary mechanisms that underlie diversification in somatic stoichiometry. Using the stoichiometric framework to understand the ecological consequences of evolutionary change requires investigation of the mechanisms underlying evolution of the elemental phenotype.

While there have been elegant studies explaining the phylogenetic diversification of elemental composition (e.g., Quigg et al. 2003), we know little about the microevolutionary mechanisms that underlie such diversification. Genetic variation is the fuel that drives evolutionary change in phenotypes. Bertram et al. (2008) found that intraspecific variation in nitrogen (N) and phosphorus (P) content of a single species of cricket was as variable as that observed across all insect taxa (Woods et al. 2004). In light of such new discoveries, particularly the substantial intraspecific variation in a few frequently studied elements in ecology (Table 1), we posit that a revised framework of ecological stoichiometry has the potential to shed light on evolution-to-ecology links. Specifically, attention to genetically based variation in the elemental phenotype (Fig. 3C) would facilitate the application of population genetic theory to model and empirically verify the variety of evolutionary mechanisms leading to rapid and striking divergence in the elemental phenotype (Fig. 3D) that can accompany diversification in classic phenotypes. It follows that stoichiometric theory can be applied to predict the ecological consequences of such divergence, similar to those applied to understand alterations to consumer-driven nutrient cycling following shifts at the community (i.e., interspecific) level. For example, Elser et al. (1988) found that factors limiting primary production shifted depending on the abundance of N-rich consumers (e.g., copepods) or P-rich consumers (e.g., *Daphnia*). Whether similar ecological consequences accompany evolutionary shifts in elemental composition at the intraspecific level remains to be seen.

**Table 1:** Intraspecific variation in elemental content among consumer taxa.

Species	Element	Number of individuals or genotypes studied	Coefficient of variation in elemental content	Source
*Rivulus hartii*	C	240	7.6	El-Sabaawi et al. (2012a)
	N	240	8.4	
	P	240	19.8	
*Gryllus texensis*	C	78	6.1	Bertram et al. (2008)
	N	78	11.76	
	P	78	23.51	
*Dreissena polymorpha*	C	10	1.04	Morehouse et al. (2013)
	N	10	17.35	
	P	10	20.40	
*Perca fluviatilis*	C	140	6.56	Vrede et al. (2011)
	N	140	7.37	
	P	140	17.72	
*Hyalella azteca* (large)	C	24	7.50	Goos et al. (2014)
	N	24	7.16	
	P	47	18.57	
*Hyalella azteca* (small)	C	14	7.56	
	N	14	16.88	
	P	16	9.10	
*Daphnia pulicaria*	C	6	4.36	Jeyasingh et al. (2009)
	N	6	12.27	
	P	6	21.43	
*Poecilia reticulata*	C	˜120	7.5	El-Sabaawi et al. (2012b)
	N	˜120	9.8	
	P	˜120	19.7	
*Poecilia mexicana*	C	223	5.46	Alba et al. (D.M. Alba, L. Arias-Rodriguez, M. Tobler, P.D. Jeyasingh, unpubl. ms.)
	N	223	4.51	
	P	303	53.04	
	S	223	9.82	

## Mechanisms Underlying Variation in the Elemental Phenotype

Intraspecific variation in elemental composition has to be driven by differential acquisition, assimilation, or allocation (AAA) of elements. Ecologists have long been interested in the relative variation in resource acquisition and resource allocation, as this was thought to be the key to understanding positive correlations among life-history traits (Reznick et al. 2000; Robinson and Beckerman 2013). This observation is paradoxical given that these traits are expected to compete for the same limiting resources resulting in trade-offs. Theory predicts that acquisition should be more variable within populations than allocation, which explains why positive correlations are often found among life-history traits (Van Noordwijk and de Jong 1986; Houle 1991), although alternative explanations to this conundrum exist (Lande 1976). For example, grasshoppers that perceive the threat of predation have higher metabolism and shift their diet (i.e., their acquisition) to C (carbon)-rich food to accommodate increased C demand (Hawlena et al. 2011). Similarly, phenotypic plasticity in life-history traits of a cricket, *Gryllus firmus*, appears to be driven primarily by genetic variation in resource acquisition (Robinson and Beckerman 2013). On the other hand, positive life-history correlations persist in *Daphnia pulicaria* despite keeping resource acquisition constant (Olijnyk and Nelson 2013). This suggests that genetic variation in resource assimilation drives differences in life-history traits in this species. Although empirical research elucidating the relative contributions of acquisition, assimilation, and allocation in determining variation in the elemental phenotype is sparse, it is clear that both genes and the environment can contribute to such variation. No doubt, evolutionary diversification in the elemental phenotype should be associated with divergent acquisition, assimilation, or allocation of elements. Together, such arguments are highly congruent with predictions arising from life-history theory largely based on energetics (Flatt and Heyland 2011).

Elemental composition has been found to exhibit high intraspecific variation in autotrophs even when supply stoichiometry is controlled (i.e., through common garden experiments), and such variation is often heritable as observed by Broadley and White (2005) in several autotrophs. Moreover, plasticity in elemental composition of autotrophs in response to elemental supply is variable across individuals, and some of this variation is genetically based (e.g., Watanabe et al. 2007). Indeed, agronomists exploit such genetic variation in elemental composition to artificially select for nutrient efficient strains. For example, genetic variation in phosphorus (P) acquisition among wheat and barley genotypes responds strongly to artificial selection on biomass production (e.g., Gahoonia and Nielsen 1996). Similarly, genetic variation in allocation of P to critical tissues such as roots affects fitness in cultivars of common bean (Miller et al. 2003). Several studies have even identified quantitative trait loci (QTL) underlying such variation (e.g., Buescher et al. 2010; Conn et al. 2012), which ultimately impinge on elemental composition (Willems et al. 2010; Norton et al. 2012). Moreover, studies have found that the performance of these QTLs in predicting biomass production is dependent upon the environment, indicating substantial gene-by-environment interactions (Zhang et al. 2009; Ding et al. 2010).

## Ecological Relevance of Variation and Subsequent Evolution in Elemental Phenotypes

Evolutionary shifts in the elemental phenotype through changes in AAA of elements should ultimately affect ecology. Exploring this frontier has the potential to illuminate the links between ecology and evolution, not only because it can affect fundamental aspects of ecology such as resource consumption and demographics, but also because evolutionary shifts in AAA will alter the return of unused elements to the environment (e.g., excretion, egestion). For example, the genetic basis of variation in excretion of N and P has been documented in two broiler chicken pedigrees that are known to differ in several traits and feed conversion efficiencies (Sasikala-Appukuttan et al. 2010). We argue that similar genetic variation in AAA in natural populations, driven by changes in environmental availability or organismal demands, may cause shifts in patterns of elemental excretion. The resulting change in unused elements returned back to the environment has the potential to alter ecosystem function with consequences for all members of the community. Such important ecological effects of evolutionary change can only be deciphered when attention is paid to controlling both the genetic and environmental sources of variation in nutrient excretion. For example, P. Roy Chowdhury & P. D. Jeyasingh (unpubl. data) used radiotracer (^33^P) methods and found that genetic variation in the physiological rates of P processing in *Daphnia* altered the P content and density of algal biomass. Correlated shifts in the processing and excretion of other elements covarying with P use may also contribute to this ecological response (P. Roy Chowdhury, K.P. Roberts, P.D. Jeyasingh, unpubl. data).

## Elemental Signatures of Evolution in Classic Traits

Because the elemental phenotype emerges from variation in classic traits, elemental phenotypes consequently should be affected by both genes and the environment and shaped by the same mechanisms and constraints that underlie evolution in classic traits. Natural selection on the elemental phenotype may be direct, if environmental availability of elements constrains elemental budgets of organisms, or indirect, if adaptive modification of classic phenotypes alters elemental demands. Resulting evolutionary shifts in the elemental phenotype can affect ecology. For example, altered supply of elements such as P due to excessive application of fertilizer has resulted in microevolutionary shifts toward decreased P use efficiency of crops (Evans 1993), indicating a shift in ecological function from a P sink to a P source.

It is likely that evolution of phenotypes that have distinctive elemental demands translates into differential use of several of the ˜25 elements required for life. All classical traits require more than one element. As such, an evolutionary shift in one trait should affect demand and processing of suites of elements. For example, reductions in the number of bony lateral plates in populations of threespine stickleback (*Gasterosteus aculeatus*; see Box 2) in freshwater ecosystems (Bell and Foster 1994) should not only result in altered P content and processing compared to marine counterparts with more lateral plates, but also in changes of calcium content and processing because bone is ˜2 parts calcium: ˜1 part P (Kramer and Shear 1928). Furthermore, such evolutionary shifts in body armor can affect processing of sodium because the cellular physiology of P transport into cells is dependent on sodium (Werner et al. 1998). The ecological consequences of such correlated shifts in the use of multiple elements (e.g., Kaspari et al. 2009) will be important for a rigorous exploration of how evolutionary shifts impact ecology.

**Box 2. *Illuminating the potential ecological consequences of phenotypic evolution in sticklebacks using the stoichiometric framework***

Armored (oceanic, top) and unarmored (freshwater, bottom) threespine stickleback (*Gasterosteus aculeatus*). Loss of bony lateral plates, made of calcium and phosphorus, should lead to lower Ca and P content in unarmored form compared to armored ancestors. 
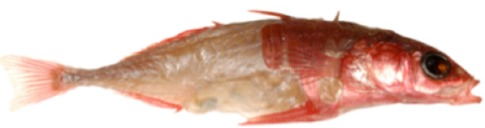
 
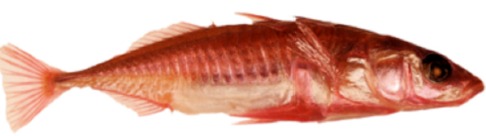

No differences in Ca and P content between the two forms indicate reallocation of Ca and P from lateral plates to other traits in the freshwater form. Such shifts in allocation of Ca and P within the individual would be ecologically neutral within the stoichiometric framework, although accommodative shifts in the content of other elements (e.g., sodium) could alter the elemental phenotype, with potentially unique shifts in ecological functions.

Alternatively, a difference in the content of Ca and P between the two forms would indicate altered rates at which Ca and P are acquired, assimilated, and/or excreted, with potentially distinct consequences at the community and ecosystem level, as shown for benthic and limnetic freshwater forms (Harmon et al. 2009).

Similar experiments with stoichiometrically explicit data collection and analyses have great potential to rigorously answer the questions furnished in Fig. 3. Photo courtesy: RDH Barrett (Barrett et al. 2008).

## Integrating Ecological Stoichiometry and Population Genetics to Investigate the Ecological Consequences of Evolutionary Change

**Figure 4:**
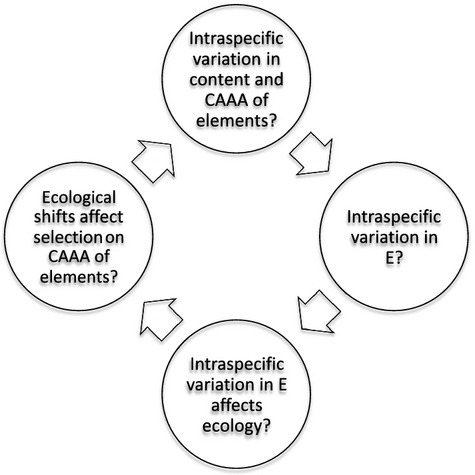
A hypothetical eco-evo-eco loop for a population based on the revised stoichiometric framework. CAAA = Content, Acquisition, Assimilation, Allocation; E = excretion of any element. Initial genetic variation in CAAA of an element can be maintained as a consequence of selection on phenotypes requiring the element, potentially impacting organismal elemental composition and excretion of the element. Altered excretion of the element can affect the supply of the element and generate ecological shifts. Such shifts can alter selection on various phenotypes that manifest as altered selection on CAAA in subsequent generations.

Several key questions have to be addressed for a formal integration of ecology and evolution using the stoichiometric framework (Fig. 4). Although there is mounting evidence for a handful of biologically relevant elements (Table 1), rigorously testing whether there is genetic variation in the elemental phenotype is important, particularly using quantitative genetic methods (see Colmsee et al. 2012). Moreover, whether shifts in the elemental phenotype lead to subsequent impacts on demographics, ecological interactions, and consumer-driven nutrient recycling needs to be verified. Note that ecological stoichiometry (Sterner and Elser 2002) is based on variation in the elemental phenotype at the species level, generating robust predictions about ecological functions of species. It is likely that similar predictions can be made at the intraspecific level, especially given that within-species variation in the elemental phenotype rivals across-species measures (Bertram et al. 2008). These studies show that genetic variation in elemental content differs considerably based on the element in question (Table 1). Hence, evolutionary changes in classic phenotypes may have distinct effects on the evolution of each of the ˜25 elemental traits that represent the elemental phenotype, perhaps with unique ecological consequences.

## Concluding Remarks

Data on intraspecific variation in the elemental phenotype are sparse, even for the three well-studied elements (C,N,P). Quantitative genetic studies on the elemental phenotype are necessary to understand its evolution. Nevertheless, even the sparse existing data (Table 1) indicate that some elemental traits (e.g., P, N) harbor more genetic variation compared to others (e.g., C). In addition, it is possible that evolution of some elemental traits is more sensitive to environmental supply. The possibility of elemental traits evolving at different rates (Table 1), due to genetic constraints and selection imposed by the environment, will be a central frontier in refining the proposed framework (Fig. 4) to decipher the ecological consequences of evolutionary change in various taxa (e.g., sticklebacks; Box 2).

Although already evident, we reiterate that the hypotheses arising from the stoichiometric framework we have proposed is not meant to replace the traditional trait-based approaches that are commonly used to study the ecological relevance of evolutionary change. We agree selection acts on traditional traits that result in evolutionary shifts that potentially affect some aspect of ecology. However, quantifying classical phenotypes comprehensively is beyond the scope of present-day technologies (Houle et al. 2010). The framework we have proposed is based on the fact that *any* evolutionary shift in a classic trait should be visible at the elemental level (i.e., in CAAA&E of ˜25 elements). As such, we have abstracted the staggering complexity of classic phenotypes into about 25 variables that are readily quantifiable in all taxa, at all levels of biological organization. We realize that our approach abstracts much of the biological complexity that underlies the poorly explored evolution-to-ecology links. However, such abstraction based on elements has a rich history in revealing fundamental ecological mechanisms (Tilman 1982; Sterner and Elser 2002). Whether the amended framework of ecological stoichiometry, one that embraces intraspecific variation in the elemental phenotype, illuminates the mechanisms underlying the ecological consequences of evolutionary change, as proposed here, remains a hypothesis. Integrating powerful approaches in ecology, such as stoichiometric theory, and evolution, such as population genetic theory, as proposed here should generate logically consistent and empirically verifiable arguments to refine our understanding of the ecological consequences of phenotypic evolution.
